# Angiotensin II Type 2 Receptor Decreases Transforming Growth Factor-β Type II Receptor Expression and Function in Human Renal Proximal Tubule Cells

**DOI:** 10.1371/journal.pone.0148696

**Published:** 2016-02-11

**Authors:** Hui-Lin Guo, Xiao-Hui Liao, Qi Liu, Ling Zhang

**Affiliations:** 1 Department of Nephrology, The Second Affiliated Hospital, Chongqing Medical University, Chongqing, 400010, China; 2 Institute for Viral Hepatitis, Key Laboratory of Molecular Biology for Infectious Diseases, The Second Affiliated Hospital, Chongqing Medical University, Chongqing, 400010, China; George Washington University School of Medicine and Health Sciences, UNITED STATES

## Abstract

Transforming growth factor-β (TGF-β), via its receptors, induces epithelial-mesenchymal transition (EMT) and plays an important role in the development of renal tubulointersitial fibrosis. Angiotensin II type 2 receptor (AT_2_R), which mediates beneficial renal physiological functions, has received attention as a prospective therapeutic target for renoprotection. In this study, we investigated the effect and underlying mechanism of AT_2_R on the TGF-β receptor II (TGF-βRII) expression and function in human proximal tubular cells (HK-2). Here, we show that the AT_2_R agonist CGP42112A decreased TGF-βRII protein expression in a concentration- and time-dependent manner in HK-2 cells. The inhibitory effect of the AT_2_R on TGF-βRII expression was blocked by the AT_2_R antagonists PD123319 or PD123177. Stimulation with TGF-β1 enhanced EMT in HK-2 cells, which was prevented by pre-treatment with CGP42112A. One of mechanisms in this regulation is associated with the increased TGF-βRII degradation after activation of AT_2_R. Furthermore, laser confocal immunofluorescence microscopy showed that AT_2_R and TGF-βRII colocalized in HK-2 cells. AT_2_R and TGF-βRII coimmunoprecipitated and this interaction was increased after AT_2_R agonist stimulation for 30 min. The inhibitory effect of the AT_2_R on TGF-βRII expression was also blocked by the nitric oxide synthase inhibitor L-NAME, indicating that nitric oxide is involved in the signaling pathway. Taken together, our study indicates that the renal AT_2_R regulates TGF-βRII expression and function via the nitric oxide pathway, which may be important in the control of renal tubulointerstitial fibrosis.

## Introduction

Renal tubulointerstitial fibrosis is often regarded as the final outcome of a wide range of progressive chronic kidney diseases and is a final common pathway to end-stage chronic kidney diseases whose severity correlates with renal prognosis [1]. Proximal tubular epithelial cells play a pivotal role in renal tubulointerstitial fibrosis. Emerging evidence suggests that a critical step in the pathogenesis of tubulointerstitial fibrosis is epithelial-mesenchymal transition (EMT), a pathological process characterized by a phenotypic conversion from epithelial cells to fibroblast-like morphology [2]. During EMT, tubular epithelial cells lose their epithelial phenotype and acquire a mesenchymal phenotype. This phenotypic conversion involves the *de novo* synthesis of mesenchymal markers such as α-smooth muscle actin (α-SMA), and a downregulation of epithelial markers such as E-cadherin that is essential for the structural integrity of renal epithelium [2,3].

It is generally accepted that of a variety of cytokines and growth factors that trigger EMT, transforming growth factor-β (TGF-β) is the major profibrotic cytokine that contributes to tubulointerstitial damage and renal fibrosis via numerous intracellular signal transduction pathways [4]. Active TGF-β initiates cell signaling by binding to its transmembrane serine/threonine kinase receptors type I (TGF-βRI) and type II (TGF-βRII). Binding of TGF-β to receptor type II leads to the recruitment and phosphorylation of receptor type I, which further activates its downstream signaling via the Smad-dependent or -independent pathways and directly leads to the initiation of EMT [4,5]. Moreover, many other cytokines such as interleukin-1 and angiotensin II (Ang II) also have effects on EMT indirectly via the induction of TGF-β [6,7]. In addition, the effects of other cytokines such as tumor necrosis factor-alpha (TNF-α) may be synergistic with that of TGF-β [8]. Since the subtypes of receptors are primarily engaged in the initial binding of TGF-β, the potent effect of TGF-β on the induction of EMT is dependent on its receptors. So how to suppress its profibrotic receptors activation-induced EMT in renal tubular epithelial cells is an important issue to prevent renal tubulointerstitial fibrosis.

Ang II, considered as the primary mediator of classic renin-angiotensin system (RAS), exerts its action by binding totwo major receptor subtypes, namely type 1 (AT_1_R) and type 2 (AT_2_R). AT_1_R mediates the major actions of Ang II, including vasoconstriction, renal tubule sodium reabsorption, inflammation, and aldosterone secretion [9,10]. However, AT_2_R is generally considered to be a functional antagonist of AT_1_R and is thought to exert beneficial effects, including promoting natriuresis, preventing fibrogenesis, lowering blood pressure, and modulating inflammation [11–13]. In recent years, studies have paid more attention to the interaction between Ang II receptors and TGF-β receptors in the cardiovascular system and kidney. Activation of AT_1_R enhances the expression of TGF-βRI [14]; but transfection of the AT_2_R gene suppresses the expression of TGF-βRI in vascular smooth muscle cells (VSMCs) [15]. In proximal tubular cells, stimulation of AT_1_R increases TGF-βRII expression [16]; however, TGF-β1 stimulation decreases AT_1_R level in VSMCs [17]. Because both AT_2_R and TGF-β receptors are well-expressed in renal proximal tubular, we hypothesize that AT_2_R may also regulate TGF-β receptors expression and function in kidney. The present study showed that activation of AT_2_R decreased TGF-βRII, not TGF-βRI, expression and function in human proximal tubular epithelial cells (HK-2). One of mechanisms of decreased TGF-βRII was associated with the increased TGF-βRII degradation after stimulation of AT_2_R. Nitric oxide is involved in the regulation of the AT_2_R on the expression of TGF-βRII. Moreover, AT_2_R/TGF-βRII colocalized and coimmunoprecipitated in HK-2 cells; both were increased by short-term stimulation of AT_2_R. Our findings suggest that the regulation of AT_2_R on TGF-βRII may be important in the control of renal tubulointerstitial fibrosis.

## Materials and Methods

### Cell Culture

The immortalized human proximal tubule epithelial cells (HK-2) were obtained from the American Type Culture Collection (Rockville, MD, USA). Cells were cultured in Dulbecco’s modified eagle medium (DMEM) (Gibco BRL, Grand Island, NY, USA) supplemented with 10% fetal bovine serum (FBS) at 37°C in a humidified incubator (5% CO_2_, 95% air). All experiments were performed in serum-free conditions. Cells were growth arrested in serum-free medium for 24 h before being used in experiments. Then the cells were incubated with TGF-β1 (5 ng/mL) or the AT_2_R agonist CGP42112A (10^−7^ M) for the indicated time points.

### Materials

TGF-β1 and CGP42112A were obtained from Sigma (St. Louis, MO, USA). The AT_2_R antagonists, PD123319 and PD123177, and cycloheximide were also obtained from Sigma. Antibodies against TGF-βRII and E-cadherin were rabbit anti-human polyclonal antibodies. The antibody against α-SMA was mouse monoclonal antibody. The antibody against AT_2_R was an affinity purified goat polyclonal antibody. All of the antibodies were obtained from Santa Cruz Biotechnology, Inc (Dallas, TX, USA). All other chemicals for various buffers were of the highest purity available and purchased exclusively from Sigma or Gibco (Gibco, Grand Island, NY, USA).

### Immunoblotting

The protein content of the cell lysates was determined using bicinchoninic acid (BCA) protein assay reagent (Pierce Biotechnology, Rockford, IL, USA). Samples containing 50 μg of cell protein were separated in 10% SDS—PAGE and transferred into nitrocellulose membranes. The membranes were then blocked with 5% non-fat milk powder in phosphate-buffered saline (PBS)-T (0.05% Tween 20 in 10 mmol/L phosphate buffered saline) for 1 h at room temperature, and then incubated overnight with the primary antibodies, including TGF-βRII (1:500), AT_2_R (1:400), α-SMA (1:600), and E-cadherin (1:400). Then the blots were washed with PBST and then incubated with secondary antibody conjugated to horseradish peroxidase for 1 h at room temperature. Detection was done with the chemiluminescence reagent (Amersham Biosciences, Piscataway, NJ, USA). The density of the bands was quantified by densitometry using the program, Quantiscan.

### Immunofluorescence staining

HK-2 cells were fixed in 4% cold paraformaldehyde for 20 min. After washing with PBS for 3 times, the fixed cells were incubated in 0.05% Triton X-100 at room temperature for 5 min, followed by incubated with 1% BSA blocking buffer at room temperature for 30 min. Subsequently, anti-α-SMA (1:200) or E-cadherin (1:150) antibody was added and incubated with cells at 4°C overnight. Then, cells were washed with PBS and incubated with fluorescein isothiocyanate—conjugated donkey anti-mouse or anti-rabbit secondary antibody at room temperature for 1 h. After washing three times with PBS, fluorescence images were obtained with a Nikon E600 Upright Epifluorescence Microscope (Nikon, Tokyo, Japan).

### Immunofluorescence confocal microscopy

Co-localization of TGF-βRII and AT_2_R was performed in cultured HK-2 cells. The cells, grown on poly-D-lysine-coated cover slips (BD Biosciences, San Jose, CA, USA), were fixed for 20 min with 4% cold paraformaldehyde, permeabilized for 5 min with 0.05% Triton X-100, and then double immunostained for TGF-βRII and AT_2_R overnight at 4°C. The AT_2_R (1:200) was visualized using an IgG affinity- purified polyclonal goat anti-AT_2_R antibody followed by a rhodamine-conjugated donkey anti-goat secondary antibody (red; Molecular Probes, OR, USA). The TGF-βRII was visualized using an IgG affinity-purified polyclonal rabbit anti-TGF-βRII antibody (1:200), followed by a fluorescein isothiocyanate—conjugated donkey anti-rabbit secondary antibody (green; Molecular Probes, OR, USA). Immunofluorescence images were obtained using Olympus AX70 laser confocal microscopy.

### Co-immunoprecipitation

Cell lysates from cells were prepared using lysis buffer supplemented with protease inhibitors (1 mM PMSF, 5 μg/ml aprotinin, and 5 μg/ml leupeptin). Equal amounts of cell lysates (500 μg protein) were incubated with AT_2_R antibody (2.0 μg) for 1 h and protein A/G plus-agarose at 4°C overnight. After washing, samples were re-suspended in Laemmli buffer, boiled for 5 min, and subsequently loaded onto SDS-PAGE gels, which were analyzed by immunoblotting. The density of the bands was quantified by densitometry using the program, Quantiscan.

### Statistical Analysis

The data are expressed as mean ± standard error of the mean (SEM). Significant difference between two groups was determined by Student’s *t*-test, while that among 3 or more groups was determined by one-way factorial ANOVA followed by Holm-Sidak post-hoc test. A *P* value < 0.05 was considered significant difference.

## Results

### AT_2_R decreases TGF-βRII expression in HK-2 cells

**Fig 1:**
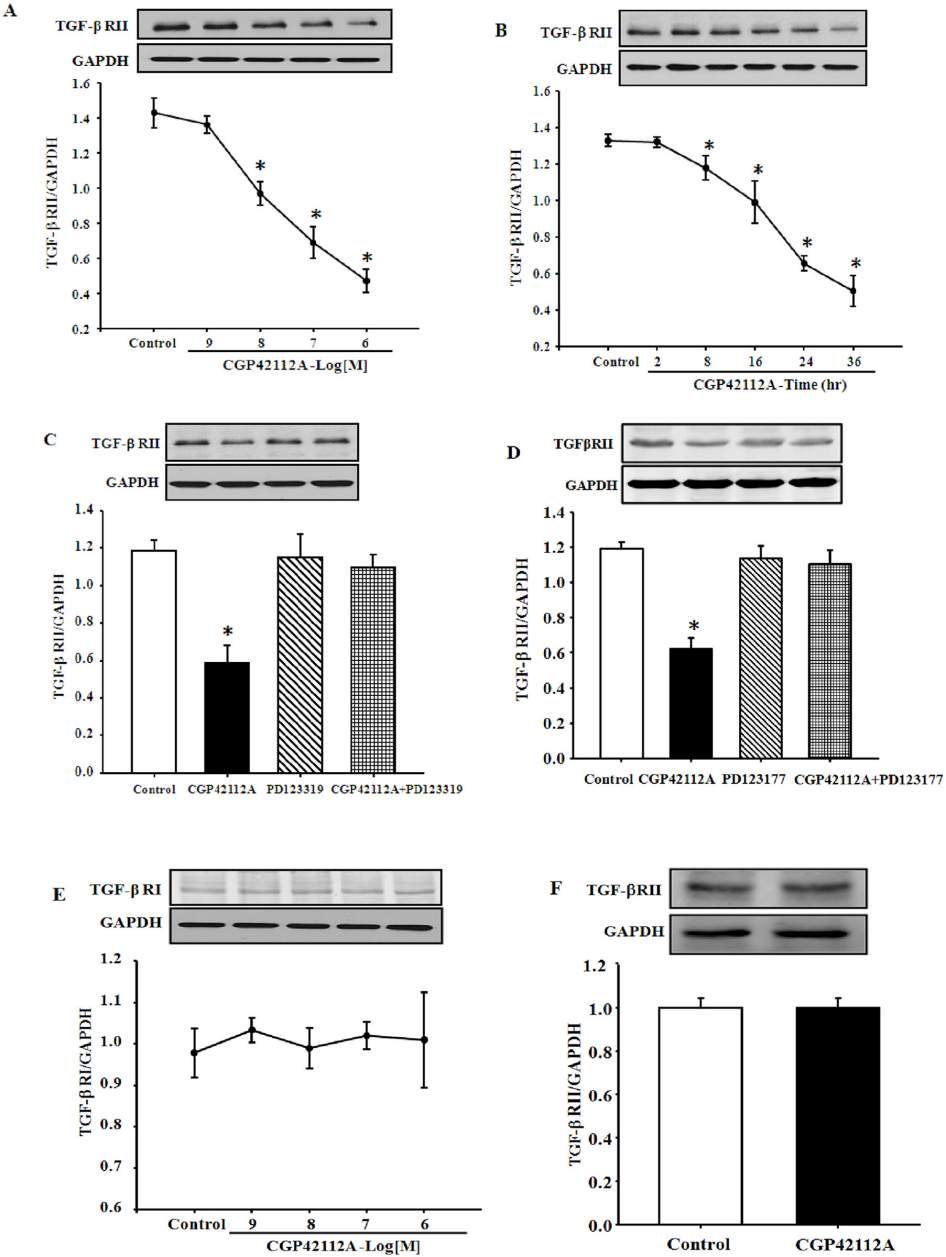
Effect of CGP42112A on TGF-βRII protein expression in HK-2 cells. (A and B) Concentration-response (24 h) (A) and time-course (10^−7^ M) (B) of TGF-βRII protein, determined by immunoblotting, in HK-2 cells treated with CGP42112A. Results are expressed as the ratio of TGF-βRII to GAPDH densities (n = 4, *P<0.05 vs. control, one-way factorial ANOVA followed by Holm-Sidak post-hoc test). (C and D) The cells were incubated with the indicated reagents (CGP42112A, 10^−7^ M; PD123319, 10^−6^ M [C]; PD123177, 10^−6^ M [D]) for 24h. Results are expressed as the ratio of TGF-βRII to GAPDH densities (n = 3–5, *P<0.05 vs. others, one-way factorial ANOVA followed by Holm-Sidak post-hoc test). (E) TGF-βRI protein expression in HK-2 cells treated with CGP42112A. Results are expressed as the ratio of TGFβRI to GAPDH densities (n = 4). (F) Effect of CGP42112A on TGF-βRII protein expression in immortalized H9c2 cardiomyocytes. The cells were incubated with CGP42112 (10^−7^ M) for 24 h. Results are expressed as the ratio of TGF-βRII to GAPDH densities (n = 6).

We determined whether or not activation of the AT_2_R causes expression changes of TGF-β receptors in HK-2 cells. Treatment with the AT_2_R agonist CGP42112A decreased TGF-βRII expression in a concentration- and time-dependent manner in HK-2 cells (Fig 1A and 1B). The inhibitory effect was significant at and >10^−8^ M (Fig 1A); the inhibitory effect of CGP42112A (10^−7^ M) was noted as early as 8 h and maintained for 36 h (Fig 1B).

The specificity of CGP42112A as an AT_2_R agonist was also determined by studying the effect of the AT_2_R antagonists, PD123319 and PD123177. Consistent with the study shown in Fig 1A, CGP42112A (10^−7^ M/24 h) decreased TGF-βRII expression. The AT_2_R antagonists PD123319 (10^−6^ M) or PD123177 (10^−6^ M) had no effect on TGF-βRII expression by themselves, but reversed the inhibitory effect of CGP42112A on TGF-βRII expression (Fig 1C and 1D).

The inhibitory effect of the AT_2_R on TGF-βRII expression was receptor-specific because stimulation of the AT_2_R had no effect on TGF-βRI expression in HK-2 cells (Fig 1E). Moreover, the effect of AT_2_R on TGF-βRII expression was also tissue- specific because in immortalized H9c2 cardiomyocytes, stimulation of the AT_2_R had no effect on TGF-βRII expression (Fig 1F).

### AT_2_R inhibits the TGF-β1-mediated EMT in HK-2 cells

**Fig 2:**
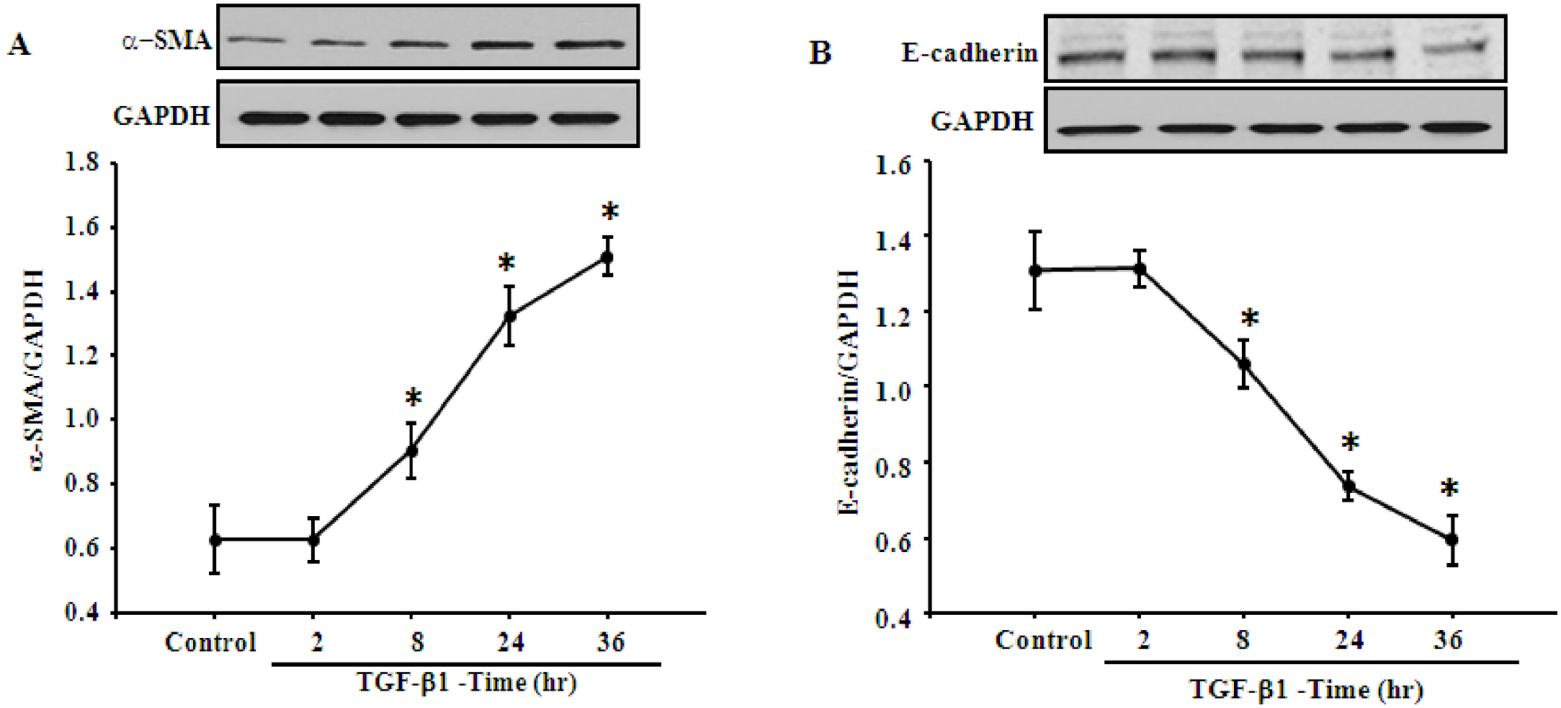
Effect of TGF-β1 on the EMT in HK-2 cells. **(A and B)** Time-response of TGF-β1 on expression of α-SMA and E-cadherin in HK-2 cells. HK-2 cells were treated with TGF-β1 (5 ng/mL) for indicated time points, and then the expression of α-SMA (A) and E-cadherin (B) were analyzed by immunoblotting. Results are expressed as the ratio of α-SMA or E-cadherin to GAPDH densities (n = 4, *P<0.05 vs. control, one-way factorial ANOVA followed by Holm-Sidak post-hoc test).

To determine the effect of TGF-β1 on EMT of renal tubular epithelial cells, HK-2 cells were treated with TGF-β1 (5 ng/mL) at various time points (2, 8, 24 and 36 h). TGF-β1 treatment increased EMT in HK-2 cells in a time-dependent manner, which resulted in the gradual increase in expression of α-SMA (Fig 2A), an important marker of myofibroblast, and decrease in expression of E-cadherin, a typical phenotypic marker of epithelial cell (Fig 2B). These demonstrate that TGF-β1 promotes EMT in normal human renal tubular epithelial cells, which is consistent with previous studies [18,19].

**Fig 3:**
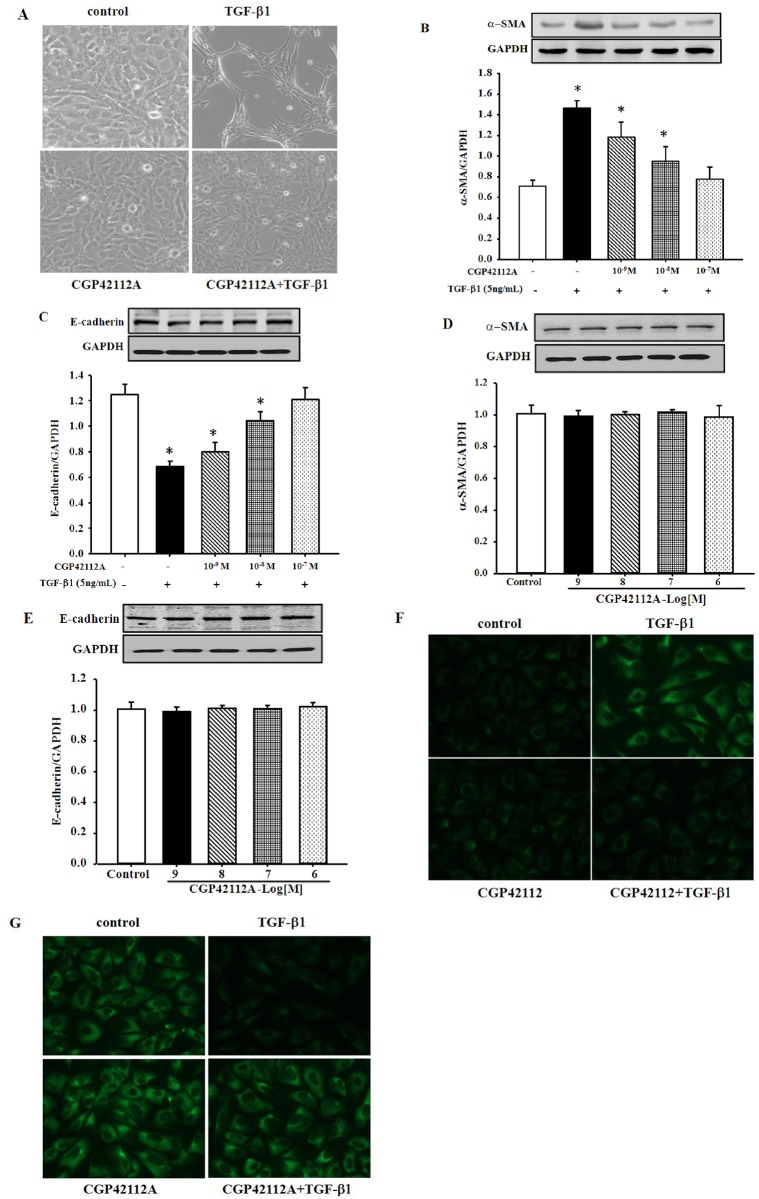
Inhibitory effect of AT_2_R on TGF-β1-mediated EMT in HK-2 cells. HK-2 cells were pretreated with CGP42112A at the indicated dose for 1 h and then treated with TGF-β1 (5 ng/mL) for 24 h. (A) CGP42112A attenuated the morphologic changes induced by TGF-β1 in HK-2 cells (Magnification 100×). (B and C) Pretreatment with CGP42112A abrogated TGF-β1-induced α-SMA expression (B) and restored E-cadherin expression (C). Results are expressed as the ratio of TGF-βRII to GAPDH densities (n = 4–5, *P<0.05 vs. control, one-way factorial ANOVA followed by Holm-Sidak post-hoc test). (D and E) Expression of α-SMA (D) and E-cadherin (E) in HK-2 cells treated with CGP42112A at the indicated dose. Results are expressed as the ratio of α-SMA or E-cadherin to GAPDH densities (n = 4). (F and G) Immuofluorescence staining of α-SMA (F) and E-cadherin (G) in HK-2 cells.

Next, HK-2 cells were incubated with TGF-β1 and/or CGP42112A for 24 h to investigate whether or not CGP4211A, an AT_2_R agonist, inhibits the EMT in renal tubular epithelial cells induced by TGF-β1. First, the morphological changes were observed. The results showed that HK2 cells treated with TGF-β1 (5 ng/mL) for 24 h underwent phenotypic conversion from epithelial cells to myofibroblast-like cells. The AT_2_R agonist CGP42112A (10^−7^ M) had no effect on the phenotypic conversion by itself, but reversed the effect of TGF-β1 on the phenotypic conversion of HK-2 cells (Fig 3A).

Second, the expression of α-SMA and E-cadherin were also determined. Consistent with Fig 2A and 2B, western blot analysis revealed that TGF-β1 increased the expression of α-SMA, but decreased that of E-cadherin. Pretreatment with CGP42112A (10^−7^ M) dramatically abrogated TGF-β1-induced α-SMA expression (Fig 3B) and restored E-cadherin expression in a dose-dependent manner (Fig 3C). However, CGP42112A (10^−7^ M) *per se* had no effect on the expression of α-SMA and E-cadherin (Fig 3D and 3E). Furthermore, the inhibitory effect of AT_2_R on the TGF-β1-induced EMT in HK-2 cells was also confirmed with evaluation of the expression of α-SMA and E-cadherin via immunofluorescence method (Fig 3F and 3G).

Moreover, we also checked the TGF-β1 levels in the cell culture media. Treatment with CGP42112A (10^−7^ M) for 24 h did not change the amount of TGF-β1 secreted by HK-2 cells (data not shown). Furthermore, we also evaluated the expression of TGF-β1 in cell lysates via immunoblotting, and observed that CGP42112A treatment did not change the protein expression of TGF-β1 in HK-2 cells (data not shown).

### AT_2_R accelerates the degradation of TGF-βRII protein in HK-2 cells

**Fig 4:**
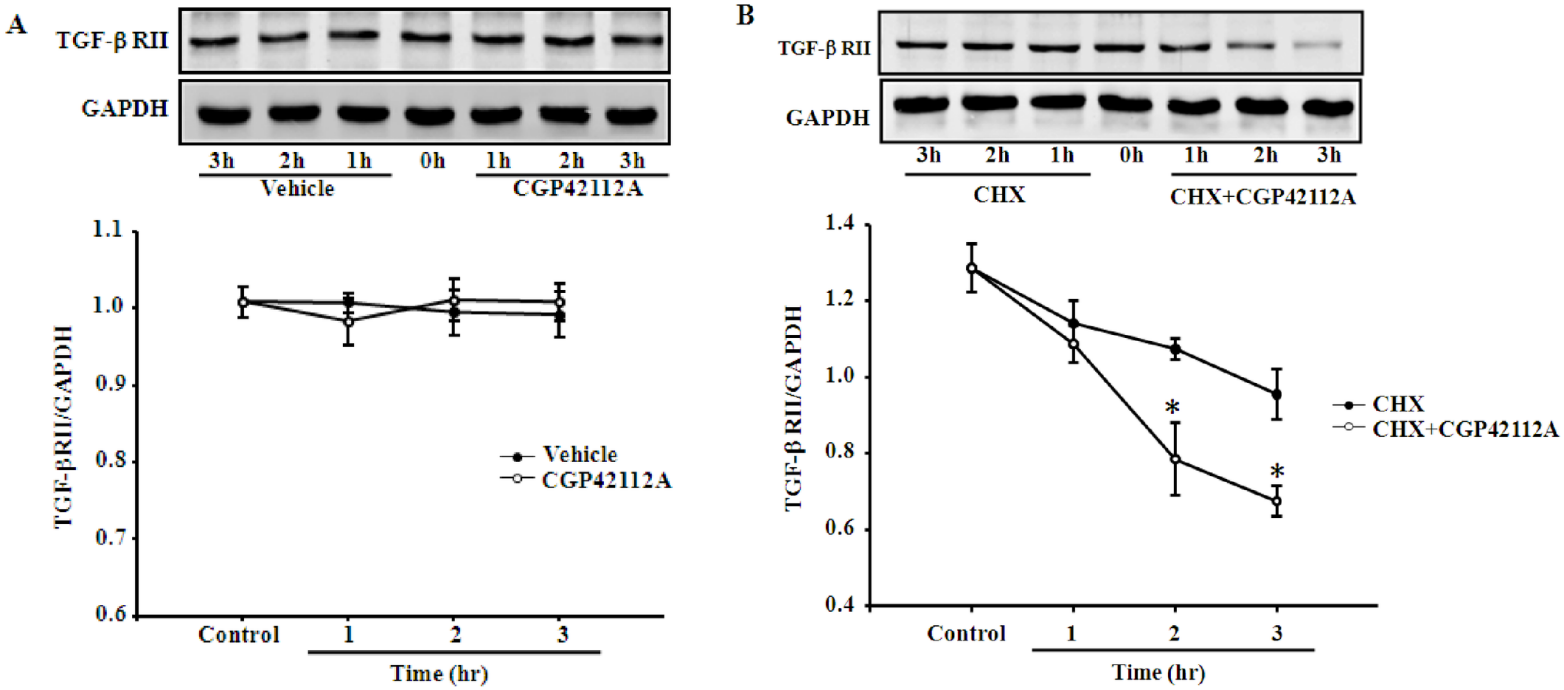
Effects of AT_2_R on TGF-βRII degradation in HK-2 cells. (A) HK-2 cells were treated with or without the AT_2_R agonist CGP42112A (10^−7^ M) for indicated time points. Results are expressed as the ratio of TGF-βRII to GAPDH densities (n = 3). (B) HK-2 cells were incubated with the AT_2_R agonist CGP42112A (10^−7^ M) with or without cycloheximide (CHX, 10 μg/mL) for indicated times. Results are expressed as the ratio of TGF-βRII to GAPDH densities (n = 3, *P<0.05 vs. cycloheximide alone, one-way factorial ANOVA followed by Holm-Sidak post-hoc test)

To elucidate the potential mechanism on the inhibitory effect of AT_2_R on TGF-βRII expression in HK-2 cells, we evaluated the TGF-βRII protein degradation levels after stimulation with AT_2_R agonist CGP42112A. We examined the TGF-βRII protein expression in the presence of 10 μg/mL cycloheximide, an inhibitor of *de novo* protein synthesis. At the indicated time, steady-state levels of TGF-βRII were examined by immunoblotting. The results showed that either vehicle or CGP42112A had no regulatory effect on TGF-βRII protein expression for up to 3 h without cycloheximide treatment (Fig 4A); however, in the presence of 10 μg/mL cycloheximide, stimulation of AT_2_R with CGP42112A (10^−7^ M) accelerated the degradation of TGF-βRII protein in HK-2 cells (Fig 4B), compared with the cells treated with vehicle. These results indicate that protein degradation is one of the mechanisms, which is involved into the regulation of AT_2_R on TGF-βRII expression in HK-2 cells.

### AT_2_R colocalizes and directly interacts with the TGF-βRII in HK-2 cells

**Fig 5:**
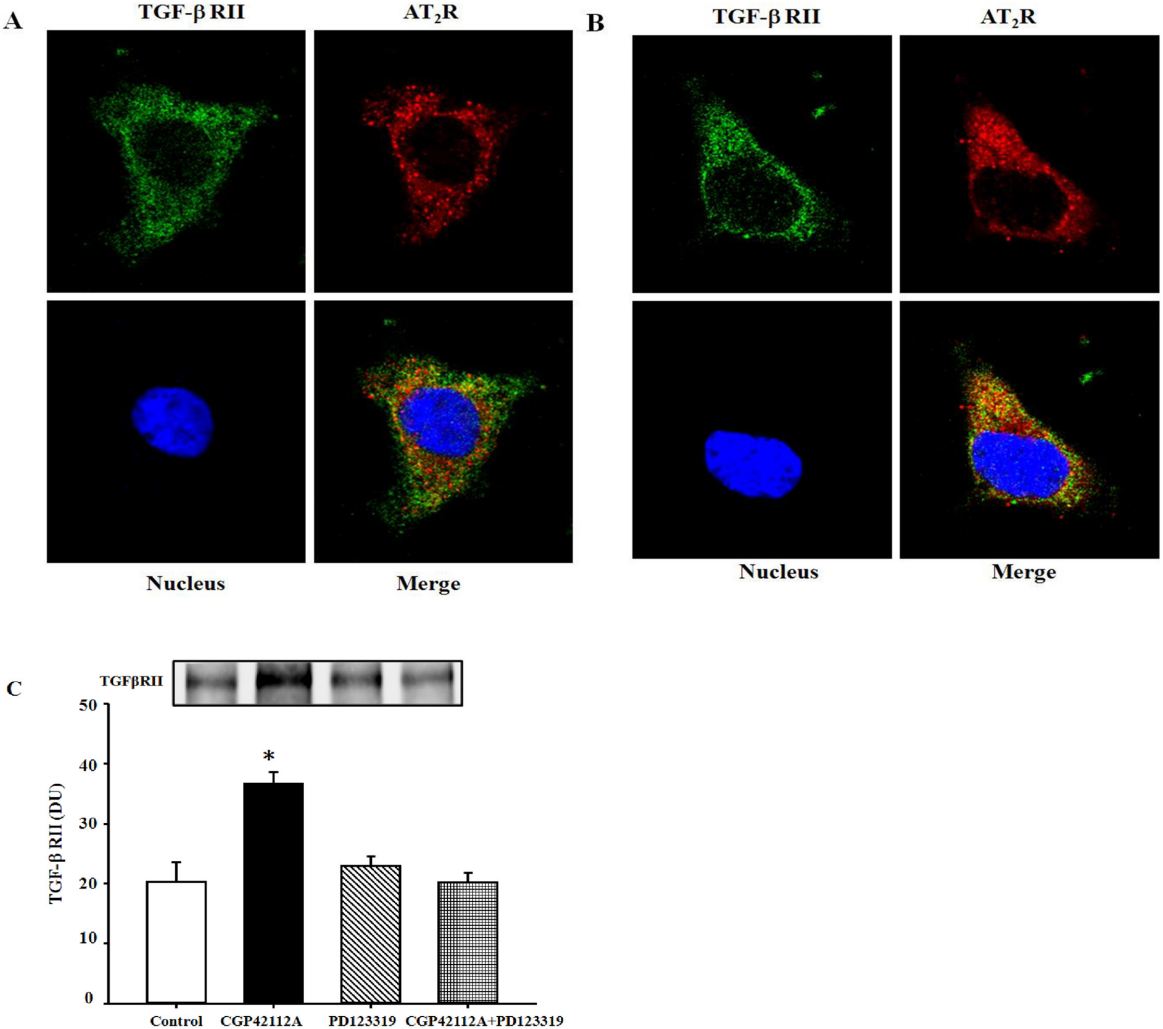
Colocalization and coimmunoprecipitation of AT_2_R and TGF-βRII in HK-2 cells. (A and B) Colocalization of AT_2_R and TGF-βRII in HK-2 cells in the basal status and after CGP42112A treatment (10^−7^ M/30 min). The cells grown on coverslips were washed, then fixed and double-immunostained for AT_2_R and TGFβRII, as described in the Methods. Colocalization appears as yellow after merging the images of fluorescein isothiocyanate—tagged TGF-βRII (green) and rhodamine-tagged AT_2_R (red). (C) Coimmunoprecipitation of AT_2_R and TGF-βRII in HK-2 cells. The cells were incubated with the indicated reagents (CGP42112A, 10^−7^ M; PD123319, 10^−6^ M) for 30 min. Thereafter, the samples were immunoprecipitated with anti-AT_2_R and immunoblotted with anti-TGF-βRII antibodies. Results are expressed as relative density units (DU) (n = 3, *P<0.05 *vs*. control, t-test).

To determine the possibility for a direct or indirect interaction between AT_2_R and TGF-βRII, we studied the colocalization of AT_2_R and TGF-βRII in HK-2 cells. Immunofluorescence laser confocal microscopy showed that AT_2_R and TGF-βRII colocalized in HK-2 cells, which is enhanced by the stimulation of AT_2_R (Fig 5A and 5B). A direct physical interaction between AT_2_R and TGF-βRII was confirmed by coimmunoprecipitation in the basal state, which was also increased following activation of the AT_2_ receptor with CGP42112A (10^−7^ M/30 min) (Fig 5C). The AT_2_ receptor antagonist, PD123319 (10^−6^ M), by itself, had no effect, but reversed the stimulatory effect of CGP42112A on the coimmunoprecipitation of AT_2_R and TGF-βRII (Fig 5C).

### Role of nitric oxide in the inhibitory effect of AT_2_R on TGF-βRII expression in HK-2 cells

**Fig 6:**
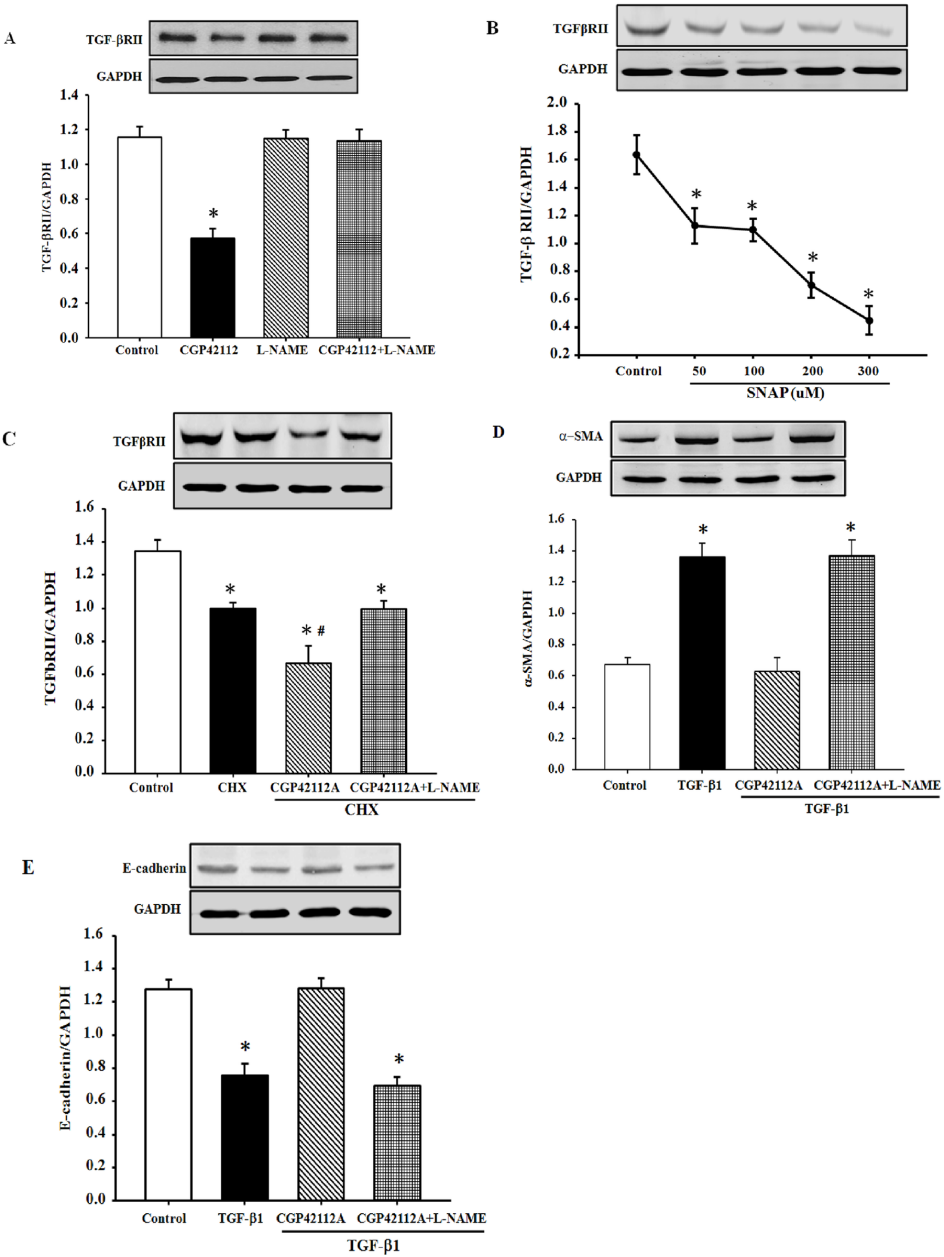
Role of nitric oxide in the inhibition of TGF-βRII expression by AT_2_R activation in HK-2 cells. (A) The cells were incubated with the indicated reagents (CGP42112A, 10^−7^ M; L-NAME, 10^−4^ M) for 24 h. Results are expressed as the ratio of TGF-βRII to GAPDH densities (n = 4, *P<0.05 vs. others, one-way factorial ANOVA followed by Holm-Sidak post-hoc test). (B) The cells were treated with different concentrations of the NO donor, S-nitroso-N-acetyl-DL-penicillamine (SNAP, 50–300 μM) for 24 h. Results are expressed as the ratio of TGF-βRII to GAPDH densities (n = 3, *P<0.05 vs. control, one-way factorial ANOVA followed by Holm-Sidak post-hoc test). (C). HK-2 cells were incubated with the indicated reagents (CGP42112A, 10^−7^ M; cycloheximide [CHX], 10 μg/mL; L-NAME, 10^−4^ M) for 3 h. Results are expressed as the ratio of TGF-βRII to GAPDH densities (n = 3, *P<0.05 vs. control, ^#^*P*<0.05 vs. cycloheximide alone, one-way factorial ANOVA followed by Holm-Sidak post-hoc test). (D and E) Expression of α-SMA (D) and E-cadherin (E) in HK-2 cells treated with the indicated reagents (CGP42112A, 10^−7^ M; TGF-β1, 5 ng/mL; L-NAME, 10^−4^ M) for 24 h. Results are expressed as the ratio of TGF-βRII to GAPDH densities (n = 3, *P<0.05 vs. control, one-way factorial ANOVA followed by Holm-Sidak post-hoc test).

Due to the involvement of nitric oxide in AT_2_R signaling [20], we next investigated the nitric oxide mechanism for the AT_2_R-mediated down-regulation of TGF-βRII expression in HK-2 cells. Results showed that the nitric oxide synthase inhibitor Nw-nitro-L-arginine methyl ester (L-NAME), by itself, had no effect on TGF-βRII expression; however, inhibition of nitric oxide production blocked the inhibitory effect of AT_2_R on TGF-βRII expression (Fig 6A). To further confirm the role of nitric oxide on the AT_2_R-mediated inhibition of TGF-βRII expression, cells were treated for 24 h with the nitric oxide donor, S-nitroso-N-acetyl-DL-penicillamine (SNAP, 50–300 μM). The results showed that SNAP decreased TGF-βRII expression in a concentration-dependent manner (Fig 6B). Furthermore, inhibition of nitric oxide production via L-NAME also blocked the degradation of TGF-βRII induced by AT_2_ activation (Fig 6C), suggesting that nitric oxide is involved in the regulation of AT_2_R on the degradation of TGF-βRII. In addition, we found that the nitric oxide synthase inhibitor L-NAME inhibited the suppressive effect of the AT_2_R on the EMT induced by TGF-β (Fig 6D and 6E). So these results suggest that nitric oxide is involved in the regulation of the AT_2_R on the expression of TGF-βRII.

## Discussion

There are several novel observations in the present study. First, we show that stimulation of AT_2_R with CGP42112A decreases TGF-βRII expression in human renal tubular epithelial cells. This effect is clearly exerted at the AT_2_R because an AT_2_R antagonist, either PD123319 or PD123177, completely blocks the effect of CGP42112A. The inhibitory effect of the AT_2_R on TGF-βRII expression is both receptor-specific and tissue-specific. Second, the interaction of AT_2_R and TGF-βRII has physiological significance in HK-2 cells since pre-treatment with CGP42112A for 24 h reversed the induction effect of TGF-β1 on the EMT. Third, AT_2_R colocalizes and coimmunoprecipitates with TGF-βRII in HK-2 cells. Moreover, stimulation of AT_2_R with CGP42112A increases the colocalization and the physical interaction between AT_2_R and TGF-βRII. Fourth, the inhibitory effect of the AT_2_R on TGF-βRII expression was blocked by the nitric oxide synthase inhibitor L-NAME, indicating that nitric oxide is involved in the signaling pathway.

Progressive renal fibrosis is thought to be the final common pathway of many kidney diseases that leads to end stage renal disease (ESRD). EMT has become widely accepted as a mechanism by which injured renal tubular cells transform into mesenchymal cells that contribute to the development of tubulointersitial fibrosis [1–3]. Accumulating evidence have demonstrated that TGF-β is the primary cytokine that drives fibrosis in kidney and other organs susceptible to fibrotic injury [21,22]. Similar to other studies [18,19], we confirmed the stimulatory effect of TGF-β1 on EMT in renal proximal tubule cells in the present study. Since the potent effect of TGF-β1 on the induction of EMT is dependent on its receptors, a reduced receptor expression may result in a decrease of TGF-β effects on tubular cells. Thus, the mechanism to inhibit TGF-β receptors-induced EMT is an important issue to resolve to prevent tubulointersitial fibrosis and to improve renal injury in patients with progressive chronic kidney disease.

AT_2_R, comprising 363 amino acids, belongs to the G protein-coupled receptor (GPCR) family[23,24]. AT_2_R is expressed well in the adult kidney primarily in the renal proximal tubules [11,25,26]. In recent years, more studies showed that AT_2_R plays a vital physiological role in the kidney. Activation of AT_2_R inhibits the activity of Na^+^-K^+^-ATPase in the proximal tubules and induces natriuresis in Sprague-Dawley rats, obese Zucker rats, and mice [11,25,26]. Stimulation of AT_2_R reduces albuminuria and prevents the diabetic nephropathy in Zucker diabetic fatty rats [27]. Proximal tubule AT_2_R activation is also anti-inflammatory by increasing IL-10 production, which offers renoprotection by preventing early inflammation-induced renal injury in obesity [28]. Chronic AT_2_R activation with CGP42112A for 2 weeks increases renal ACE2 activity, and attenuates AT_1_R function and blood pressure in obese Zucker rats [29]. As mentioned above, renal AT_2_R has received more attention as a prospective therapeutic target for renoprotection in patients with progressive chronic kidney disease.

There is increasing evidence for interaction between AT_2_R and other receptors in the kidney and cardiovascular system. Activation of AT_2_R with CGP42112A decreases AT_1_R expression and function in renal proximal tubule cells from Wistar-Kyoto (WKY) rats, but increases the expression of Mas receptor in HK-2 cells [29,30]. AT_2_R also downregulates AT_1_R and TGF-βRI in VSMCs from WKY rats [15,31]. AT_2_R interacts with renal dopamine receptors (DR) such as D_1_R[32,33]. Our current study shows that the AT_2_R agonist CGP42112A decreases TGF-βRII expression in human renal proximal tubule cells. This regulation is functionally relevant because pre-treatment with CGP42112A attenuates TGFβ1-induced EMT in HK-2 cells. Because CGP42112A does not change the TGF-β1 levels in the cell culture medium and cell lysates, we suggest that the decrease of TGF-βRII expression, not the TGF-β1 *per se*, is responsible for the attenuated TGF-β1-mediated EMT in CGP42112A-treated cells. It should be noted that CGP42112 has anti-inflammatory properties by binding to a yet uncharacterized binding site other than the AT_2_R [34], which may be distributed widely, although there is no report in HK-2 cells. Moreover, because our data is only limited in cell experiments, we did not study the physiological and/or pathophysiological correlates of TGF-β *in vivo* in the present study. TGF-β has many functions in the kidney, such as inducing renal fibrosis, mediating mesangial cell dysfunction, inducing autophagy and promoting apoptosis in renal tubular epithelial cells [4,35,36]. Inhibition of TGF-β receptors blocks TGF-β-induced EMT and decreases renal fibrosis [37]. So suppressing TGF-β-induced EMT in renal tubular epithelial cells has important physiological and/or pathophysiological significance.

Studies have showed that Ang II receptors, including AT_2_R, interact with TGF-β receptors in the cardiovascular system and kidney. Ang II, via AT_1_R, increases the binding of TGF-β with upregulation of TGF-βRI in VSMCs from WKY rats [14]. Stimulation of AT_1_R also stimulates protein expression of TGF-βRII, but not TGF-βRI, in mouse proximal tubular cells [16]. TGF-β1 stimulation increases the expression of AT_2_R in myoblasts and mouse skeletal muscle, but decreases AT_1_R expression in VSMCs [17,38]. Transfection of AT_2_R gene suppresses the expression of TGF-βRI in VSMCs [15]. AT_2_R also decreases the expression and function of AT_1_R in renal proximal tubular cells and VSMCs [30,31]. In the present study, we found that activation of AT_2_R attenuates TGF-βRII expression and its mediated function in HK-2 cells. It is possible that the pathological process of EMT in renal proximal tubular cells may be the result of a perturbation of the interaction among TGF-β receptors, AT_1_R, and AT_2_R, among others. However, there is a limitation of our experiment in selecting appropriate pharmacological agents because there are no compounds exclusively selective for a GPCR receptor. In the present study, we just tried our best to use the agonist and antagonist that are available in the market and have been previously used in similar experiments [25,26].

In our present study, we found that one of mechanisms of decreased TGF-βRII is associated with the increased TGF-βRII degradation after stimulation of AT_2_R. However, we cannot exclude the possibility that a decrease in TGF-βRII mRNA expression and protein synthesis may also occur. In addition, nitric oxide is involved in the AT_2_R-mediated physiological functions such as inhibiting proximal tubule sodium pump activity and inducing renal renin inhibition [26,39]. Studies have also showed that nitric oxide is involved in the protein expression in different levels [40,41]. Exposure to nitric oxide increases the protein degradation, which can be prevented by inhibiting NO with its scavenger or nitric oxide synthase inhibitor [41,42]. Our results showed that inhibition of nitric oxide production blocked the inhibitory effect of AT_2_R on TGF-βRII expression. The nitric oxide donor, SNAP, decreased TGF-βRII expression in a concentration-dependent manner. Furthermore, inhibition of nitric oxide production also blocked the AT_2_R-inducing TGF-βRII degradation. So these results suggest that the nitric oxide pathway is involved in the regulation of the AT_2_R on the expression of TGF-βRII.

In summary, we have demonstrated that the AT_2_R downregulates the expression of TGF-βRII in human proximal tubule cells. The regulation of the TGF-βRII by the AT_2_R has physiological significance. Pre-treatment of HK-2 cells with an AT_2_R agonist for 24 h reduces TGF-β1-induced EMT. AT_2_R and TGF-βRII directly interact that is enhanced by stimulation of AT_2_R. Besides the regulation of the direct protein-protein interaction, activation of the AT_2_R also accelerates the degradation of TGF-βRII protein in HK-2 cells. The nitric oxide pathway is involved in the regulation of the AT_2_R on the expression of TGF-βRII. This study reveals a possible underlying mechanism of the renal protective effects of AT_2_R, and may provide a potential candidate to renal fibrosis therapy.
